# Postoperative hypoalbuminemia and outcomes of pediatric liver transplantation

**DOI:** 10.1186/s12887-024-04831-x

**Published:** 2024-06-12

**Authors:** Alina Uasuwannakul, Chatmanee Lertudomphonwanit, Nattachai Anantasit, Pornthep Tanpowpong, Songpon Getsuwan, Chollasak Thirapattaraphan, Suporn Treepongkaruna

**Affiliations:** 1 Department of Pediatrics, Faculty of Medicine Ramathibodi Hospital, Mahidol University, Bangkok, Thailand; 2 Division of Gastroenterology, Department of Pediatrics, Faculty of Medicine Ramathibodi Hospital, Mahidol University, 270 Rama VI Road, Bangkok, 10400 Thailand; 3 Ramathibodi Excellence Center for Organ Transplantation, Faculty of Medicine Ramathibodi Hospital, Mahidol University, Bangkok, Thailand; 4 Division of Pediatric Critical Care, Department of Pediatrics, Faculty of Medicine Ramathibodi Hospital, Mahidol University, Bangkok, Thailand; 5 Division of Pediatric Surgery, Department of Surgery, Faculty of Medicine Ramathibodi Hospital, Mahidol University, Bangkok, Thailand

**Keywords:** Serum albumin, Acute kidney injury, Postoperative outcomes, Pediatric, Liver transplantation

## Abstract

**Background:**

Hypoalbuminemia after liver transplantation (LT) is associated with acute kidney injury (AKI) and poor outcomes in adult LT recipients. This study was performed to examine the association between the postoperative serum albumin level and early postoperative outcomes of LT in children.

**Methods:**

This single-center retrospective review involved pediatric LT recipients (0–18 years old) treated from January 2013 to June 2020. All patients were admitted to PICU and received standard post-LT care protocol. We divided patients into low (< 30 g/L) and normal (> 30 g/L) groups based on postoperative albumin day 1 to 3.

**Results:**

Among 108 LT recipients, most had biliary atresia. The median age at the time of LT was 1.8 years [interquartile range (IQR), 1.5–5.7]. There were 18 patients in low albumin group [median albumin level, 27.9 g/L (IQR, 25.8–29.6) and 90 patients in normal albumin group [median albumin level, 34.5 g/L (IQR, 32.4–36.9). The low albumin group had significantly higher incidence of AKI, occurring in 20% of patients with a median onset of 2.5 days following LT (IQR, 1–5). Postoperative hypoalbuminemia (OR, 4.94; 95% CI, 1.32–18.47; *p* = 0.01) and a longer operative time (OR, 1.37; 95% CI, 1.01–1.47; *p* = 0.02) were independent risk factors for AKI by multivariable analysis. No significant differences between the two groups were found in other early postoperative outcomes.

**Conclusion:**

Postoperative hypoalbuminemia was associated with early postoperative AKI following LT in children but not with other worsening outcomes.

## Background

Liver transplantation (LT) is a curative treatment for end-stage liver disease and fulminant liver failure. During the past decades, this operation has been developed resulting in excellent survival in children with chronic liver disease. Several factors, including the serum albumin level, have been studied to improve the outcomes of pediatric LT.

Albumin, the most abundant plasma protein, is synthesized in the liver and secreted into the bloodstream. Serum albumin plays an important role in the maintenance of intravascular oncotic pressure, which helps prevent fluid leakage that may result in edema, ascites, and pleural effusion. Moreover, albumin transports hormones, long-chain fatty acids, ions, and several drugs through the bloodstream. It also functions as an antioxidant, anticoagulant, and plasma buffer to maintain physiologic pH [1]. Hypoalbuminemia generally develops in the early period after LT in children. The main pathogenic mechanisms underlying this condition are redistribution of albumin secondary to extensive inflammation after major surgery, increased capillary permeability, and leakage of albumin into the interstitial space [2, 3]. Increased albumin catabolism in the postoperative period also leads to hypoalbuminemia [1]. A prospective study of adults undergoing major abdominal surgery showed that a > 10-g/L decrease in the serum albumin level on postoperative day (POD) 1 was associated with an increased risk of overall postoperative complications [4]. Similarly, patients with critical illness have dramatically increased capillary permeability and altered albumin exchange between the intravascular and extravascular compartments [5]. However, the benefit of maintaining a normal albumin level in critically ill and post-transplant patients is controversial [2, 6–11].

Several studies have focused on serum albumin and the outcomes of LT [7–11]. A retrospective study of adults who underwent living-related donor LT showed that early postoperative hypoalbuminemia (< 30 g/L) was associated with acute kidney injury (AKI), which led to other negative outcomes such as a longer intensive care unit stay and higher overall mortality [7]. A prospective study showed no difference in the postoperative course and complications between patients who underwent LT with and without albumin infusion [9]. However, data regarding the impact of serum albumin levels on oucomes in pediatric LT recipients are limited. Our study aimed to investigate the association between postoperative serum albumin levels and early outcomes, including postoperative complications, AKI, ICU stay and mortality, in pediatric LT recipients. By examining these outcomes, we aimed to contribute to the ongoing discussion and provide insights into the management of pediatric LT recipients.

## Patients and methods

### Study population

**Fig. 1:**
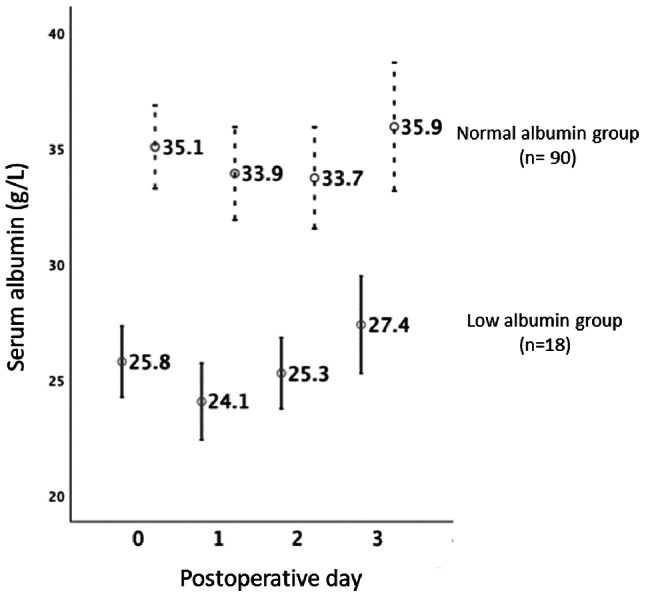
Serum albumin levels from postoperative days 1 to 3. Serum albumin levels from postoperative days 1 to 3 of the normal and low albumin groups were totally apart. The median serum albumin level in the low albumin group (*n* = 18) was 27.9 g/L (IQR, 25.8–29.6), and that in the normal albumin group (*n* = 90) was 34.5 g/L (IQR, 32.4–36.9) (*p* < 0.001)

This was a retrospective study performed in a tertiary care referral center. Medical records of all pediatric LT recipients (0–18 years of age) admitted to the pediatric intensive care unit (PICU) from January 2013 to June 2020 were reviewed. All pediatric LT recipients were admitted to the PICU immediately after LT and received a standard immunosuppression regimen (methylprednisolone and tacrolimus with or without mycophenolate mofetil). Blood tests were performed every 8 h from POD 1 to 3, every 12 h from POD 4 to 7, and then once daily up to POD 14. Fluid intake and output (abdominal drainage and urine) were recorded hourly during the first 24 h post-LT and then every 2 to 4 h until discharge from the PICU. The amount and type of intravenous fluid and blood component transfusion were adjusted according to the patients’ volume status and clinical and laboratory results. Acetar or 5% albumin was given to replace fluid loss, as per the discretion of the critical care staff, based on the amount of ascitic fluid via the drainage tube and/or the serum albumin level. A clinical pharmacist inspected the administration of all medications to avoid drug interactions and minimize nephrotoxicity. We divided the patients into two groups according to the serum albumin level on POD 1 to 3: the low albumin group (serum albumin level of < 30 g/L, *n* = 18) and the normal albumin group (serum albumin level of > 30 g/L, *n* = 90) (Fig. 1). The cutoff value of < 30 g/L for serum albumin was chosen based on clinical practice and previous study indicating that hypoalbuminemia at this levels is associated with adverse outcomes.

### Clinical data

Demographic data [age, sex, weight, length/height, underlying diseases, Pediatric End-Stage Liver Disease (PELD) score at the time of LT, and type of LT], intraoperative data (surgical time, estimated blood loss, amount and type of blood component transfusion, graft weight–recipient ratio, and fluid intake and output), postoperative complications, and mortality (up to 30 days post-LT) were reviewed. Postoperative data obtained during PICU admission included vital signs, fluid intake and output, amount of ascites (mL/kg/day), amount of albumin infusion (mL/kg/day), and Pediatric Risk of Mortality III score. Preoperative and postoperative laboratory indices (serum albumin, blood urea nitrogen, creatinine, complete blood count, coagulation indices, and tacrolimus level) were retrieved from the hospital electronic laboratory database from the day before the operation (baseline) to 7 days post-LT.

### Definitions of variables and outcomes

Postoperative AKI (from POD 1 to 3) was defined according to Kidney Disease: Improving Global Outcomes (KDIGO) 2012 [12].

The percent fluid overload, which reflected the patients’ volume status and fluid balance, was calculated using the following formula [13, 14]:

Percent fluid overload = [fluid intake (L) - fluid output (L) / PICU admission weight (kg)] × 100.

The net fluid balance was defined as the difference between total fluid intake and total output (blood loss and urine).

Persistent drainage was defined as the need for an abdominal catheter for ascites drainage for longer than 4 weeks [15].

Massive ascites was defined as more than 20 mL/kg/day of ascites from abdominal drainage from POD 1 to 7 [16].

Vascular complications were defined as any stenosis or thrombosis of the hepatic artery, portal vein, or hepatic vein requiring surgical or radiological intervention.

The vasoactive inotropic score refers to the amount of inotropes used for cardiovascular support. The score was calculated as follows [17]: dopamine dose (mcg/kg/min) + dobutamine dose (mcg/kg/min) + 100 x epinephrine dose (mcg/kg/min) + 10 × milrinone dose (mcg/kg/min) + 10,000 × vasopressin dose (U/kg/min) + 100 × norepinephrine dose (mcg/kg/min).

### Study outcomes

We compared postoperative outcomes between the low and normal albumin groups. The primary outcomes were postoperative AKI and 30-day mortality. The secondary outcomes were the length of PICU and hospital stays, duration of mechanical ventilation, need for vasopressors, use of diuretics, persistent drainage, massive ascites, intra-abdominal infection, septicemia, and vascular complications.

The study was approved by the Ethics Committee of the Faculty of Medicine Ramathibodi Hospital, Mahidol University (ID MURA2020/973).

### Statistical analysis

All data were analyzed using SPSS Version 27.0 (IBM Corp., Armonk, NY, USA). Statistical significance was defined as *p* < 0.05. Kolmogorov–Smirnov’s test was used to verify the normal distribution of continuous data. Baseline characteristics and outcome data are presented as mean ± standard deviation, median [interquartile range (IQR)], and percentage. Continuous data were compared using Student’s t-test and median test. Categorical data were compared by the chi-square test or Fisher’s exact test. We then performed univariable and multivariable logistic regression analyses to study associated factors in patients with and without AKI. Variables included in the multivariable analysis were chosen based on their clinical relevance and previous research indicating their association with AKI post-LT. The following potential risk factors for AKI were included in the multivariable analyses: preoperative and postoperative serum albumin levels, PELD score, intraoperative blood loss, operative time, use of diuretics and inotropic drugs, tacrolimus level, and septicemia.

## Results

In total, 109 children underwent LT during the study period. All children were isolated LT recipients. One patient who died intraoperatively was excluded. Thus, 108 pediatric LT recipients were enrolled in this study.

### Patients’ demographics

Of the 108 patients, 84 (78%) had underlying biliary atresia. The median age at the time of LT was 1.8 years (IQR, 1.5–5.7). A total of 100 (93%) patients underwent living-related donor LT with a median PELD score of 18 (IQR, 14–22). The median serum albumin level from POD 1 to 3 in the low and normal albumin groups was 27.9 g/L (IQR, 25.8–29.6) and 34.5 g/L (IQR, 32.4–36.9), respectively (*p* < 0.001) (Fig. 1). The patients’ demographic, preoperative, and perioperative data are summarized in Table 1.

**Table 1:** Baseline characteristics of liver transplant recipients in the low and normal albumin groups

	Total population (n = 108)	Low albumin (n = 18)	Normal albumin (n = 90)	p-value
**Preoperative variables**				
Age, years	1.8 (1.2–2.9)	2 (1.5–5.7)	1.7 (1.2–2.8)	0.23
Male sex	53 (49.1)	9 (50.0)	44 (48.9)	0.93
Body weight, kg	8.9 (7.4–12.1)	11.3 (8.1–15.7)	8.7 (7.3–11.4)	0.06
Preoperative albumin, g/L	24.7 (20.9–29.7)	24.1 (21.7–27.9)	24.7 (20.8–30.7)	0.46
PELD score	18 (14–22)	20 (15–26)	18 (14–22)	0.43
Underlying diseases				0.2
Biliary atresia	84 (77.8)	17 (94.4)	67 (74.4)	
Acute liver failure	6 (5.6)	0 (0.0)	6 (6.7)	
Others	18 (16.6)	1 (5.5)	17 (18.8)	
**Perioperative variables**				
Operative time, hours	10.3 (9.3–12)	10.1 (8.3–11.5)	10.4 (9.3–12.1)	0.29
Blood loss, mL/kg	212.4 (102.3–411.6)	186.3 (70.6–440.3)	224.4 (117–397.6)	0.49
LDLT/ DDLT	100 (92.6) / 8 (7.4)	16 (88.9) / 2 (11.1)	84 (93.3) / 6 (6.6)	0.51
Graft weight–recipient ratio	2.4 (1.9–2.8)	2.2 (1.9–2.8)	2.4 (1.9–2.8)	0.93
Intraoperative net fluid balance, mL/kg	129.4 (55.5–240.6)	76.9 (41.2–185.1)	144.3 (63.2–244.4)	0.17
**Postoperative variables**				
Pediatric Risk of Mortality Score	7 (5–7)	7.1 (4.4–8.2)	7.3 (5.2–7.6)	0.76
Maximum trough tacrolimus level, ng/mL	9.6 (6.9–12.8)	9.3 (6.9–12.8)	9.7 (6.8–12.8)	0.66

Data are presented as median (interquartile range) or n (%)

*Abbreviations* PELD, Pediatric End-Stage Liver Disease; LDLT, living-related donor liver transplantation; DDLT, deceased-donor liver transplantation

### Post-LT outcomes

The early postoperative outcomes of LT according to the postoperative serum albumin level are shown in Table 2. The incidence of postoperative AKI was significantly higher in the low than normal albumin group [*n* = 8 (44%) vs. *n* = 18 (16%), respectively; *p* = 0.005], while there were no significant differences in 30-day mortality and other outcomes/complications between the low and normal albumin groups.

**Table 2:** Outcomes of liver transplantation according to postoperative serum albumin level

Variables	Low albumin (n = 18)	Normal albumin (n = 90)	p-value
Postoperative acute kidney injury	8 (44.4)	14 (15.6)	0.005
30-day mortality	1 (5.6)	1 (1.1)	0.21
Length of PICU stay, days	10.5 (5.6–21.0)	8.3 (5.5–15.3)	0.46
Length of hospital stay, days	49 (32.5–74.3)	40.5 (30–61.2)	0.42
Duration of mechanical ventilation, days	4 (2.8–6.3)	4 (3–5)	0.92
Vasoactive-inotropic score	2.7 (0–5.8)	2.2 (2–3.7)	0.44
Albumin infusion, mL/kg/day	22.8 (9.6–48.8)	34.3 (1.0–56.3)	0.65
Use of inotropes	11 (61.1)	71 (78.9)	0.11
Massive ascites	16 (88.9)	87 (96.7)	0.15
Persistent drainage	8 (44.4)	36 (40.0)	0.73
Amount of ascites, mL/kg/day	45.6 (22.2–93.1)	46.6 (29.7–71.5)	0.79
Fluid overload	13 (72.2)	70 (77.7)	0.61
Abdominal infection	11 (61.1)	50 (55.6)	0.66
Septicemia	3 (16.7)	11 (12.2)	0.61
Use of diuretics, mg/kg/day	1.0 (0.3–1.7)	0.7 (0–1.8)	0.52
Hepatic artery thrombosis	1 (5.5)	11 (12.2)	0.41
Portal vein thrombosis	2 (11.1)	4 (4.4)	0.26
Hepatic vein thrombosis	1 (5.5)	4 (4.4)	0.84

Data are presented as median (interquartile range) or n (%)

*Abbreviation* PICU, pediatric intensive care unit

### Post-operative AKI and associated factors

Since we found that AKI was the only outcome significantly different between the normal and low albumin groups, we further analyzed the associated factors with AKI. Of all 108 patients, 22 (20%) developed AKI postoperatively. The median onset of AKI was at 2.5 days (IQR, 1–5). Twelve patients (55%) had AKI stage 1, and 10 patients (45%) had AKI stage 2. No patients had chronic kidney disease or hepatorenal syndrome, and all patients’ baseline blood urea nitrogen level, creatinine level, and urinalysis results were within normal limits before the operation. Factors that were significantly associated with AKI included a longer operative time, sepsis, and postoperative hypoalbuminemia (Table 3). Multivariable analysis with adjusted clinical variables, including preoperative albumin level, preoperative international normalized ratio, serum creatinine level, blood loss, septicemia, diuretic use, tacrolimus level, and percent fluid overload, confirmed that postoperative hypoalbuminemia (odds ratio, 4.94; 95% confidence interval, 1.32–18.47; *p* = 0.02) and a longer operative time (odds ratio, 1.37; 95% confidence interval, 1.08–1.75; *p* = 0.01) were independent risk factors for AKI (Table 4). More patients with than without AKI had septicemia (27.3% vs. 9.3%, respectively; *p* = 0.03). All patients with AKI recovered, and none required renal replacement therapy.

**Table 3:** Factors associated with postoperative acute kidney injury in pediatric liver transplant recipients

Variables	Non-AKI (n = 86)	AKI (n = 22)	p-value
Preoperative albumin, g/L	24.6 (20.2–29.1)	25.4 (23.1–31.9)	0.08
PELD score	18 (15–23)	20 (9–22)	0.58
Blood loss, mL/kg	270 (123–437)	157 (64–317)	0.07
Operative time, hours	10 (9.2–11.3)	12 (11–13.3)	0.005
Intraoperative hypotension	3 (3.5)	0 (0.0)	0.38
Intraoperative urine output, mL	32.1 (21.9–52.0)	33.9 (20.2–54.8)	0.98
Percent fluid overload	13 (1–25)	7 (7 to 17)	0.20
Use of inotropes	66 (76.7)	16 (72.7)	0.28
Use of diuretics	82 (95.3)	21 (95.5)	0.30
Septicemia	8 (9.3)	6 (27.3)	0.03
Postoperative hypoalbuminemia	14 (16.3)	8 (36.4)	0.005
Maximum trough tacrolimus level, ng/mL	11.3 (8.6–16.0)	14.3 (10.5–18.7)	0.08

Data are presented as median (interquartile range) or n (%)

*Abbreviations* AKI, acute kidney injury; PELD, Pediatric End-Stage Liver Disease

**Table 4:** Univariable and multivariable analysis of factors associated with postoperative acute kidney injury

Variables	Univariable analysis			Multivariable analysis		
	OR	95% CI	P-value	OR	95%CI	P-value
Preoperative albumin (g/L)	1.09	1.00-1.20	0.05	1.04	0.99–1.09	0.07
Preoperative INR	1.93	0.95–3.93	0.04	2.14	0.88–5.22	0.09
Operative time (hours)	1.27	1.01–1.61	0.005	1.37	1.08–1.75	0.01
Blood loss (ml/kg)	1.00	1.00-1.10	0.07	1.00	0.99-1.00	0.57
Postoperative hypoalbuminemia, n (%)	3.46	1.00-11.96	0.005	4.94	1.32–18.47	0.02
Serum creatinine (mg/dL)	3.71	2.35–37.60	< 0.001	2.69	1.11–36.20	0.05
Septicemia, n (%)	0.49	0.12–2.02	0.03	1.96	0.07–1.57	0.16
Use of diuretics, n (%)	0.16	0.01–0.26	0.99	0.95	0.04–34.18	0.54
% Fluid overload	0.97	0.96–1.01	0.32	3.20	0.83–12.35	0.09
Maximum trough tacrolimus level (ng/mL)	0.96	0.67–1.37	0.82	0.42	0.85–1.09	0.52

*Abbreviation* INR, international normalized ratio

## Discussion

In this study, we found that early postoperative hypoalbuminemia was associated with AKI in pediatric LT recipients. No other short-term clinical outcomes, including 30-day mortality, were associated with the postoperative serum albumin level. The incidence of AKI in the early period post-LT was 20%, and independent risk factors included postoperative hypoalbuminemia and a longer operative time.

A meta-analysis of adult LT recipients showed that a serum albumin level of less than 30–35 g/L was associated with AKI after LT [18]. However, a randomized controlled trial revealed no benefit of maintaining the serum albumin level above 30 g/L following LT [9]. Notably, most studies assessed the pre-transplant serum albumin level and mainly involved adults. Only one large retrospective study showed that an early postoperative serum albumin level of less than 30 g/L was an independent risk factor for AKI in adult LT recipients (similar to our study) and was associated with a higher mortality rate and longer hospitalization [10]. Hypoalbuminemia is common before LT because of the impaired synthetic function of the cirrhotic liver. The albumin level may be altered intraoperatively and postoperatively secondary to capillary leakage, blood loss, exogenous albumin infusion, and fluid balance. Net leakage of albumin from plasma progresses from the end of the surgery and persists until POD 3 [11]. Thus, we classified the patients’ albumin levels within 3 days of surgery and compared their early post-LT outcomes. Interestingly, the results of this study showed that pediatric LT recipients with a low postoperative serum albumin level were more likely to develop AKI in the early postoperative period. Moreover, when analyzing patients with and without AKI, this finding was confirmed by multivariable analysis.

AKI reportedly occurs in 18–46% of pediatric LT recipients [19–22]. Our findings revealed quite a low incidence of AKI (20%), and most cases were mild (stages 1 and 2). A recent study of pediatric LT recipients showed a 38% (43/112) incidence of AKI in the first 7 days post-LT (40% stage 1, 28% stage 2, and 32% stage 3) [19]. The reason for the lower incidence and milder degree of AKI in our study may have been the differences in demographics, underlying liver disease, baseline renal function and local protocols for perioperative management. For example, our center employs strategies such as noninvasive monitoring of the fluid status, close monitoring of the tacrolimus levels, and critical inspection of drug administration by the clinical pharmacist in the PICU. In previous studies, factors associated with post-LT AKI in children included a history of AKI prior to transplantation [19], an elevated post-LT total bilirubin level, blood loss during surgery [20], a lower preoperative serum albumin level [23], and a prolonged international normalized ratio [21]. The authors postulated that the severity of preoperative synthetic liver function may be associated with the development of postoperative AKI [21]. In this study, we found no association between AKI and the severity of liver disease as assessed by the PELD score.

The present study identified postoperative hypoalbuminemia, a modifiable factor, as an independent risk factor for post-LT AKI in children. Lower serum albumin levels can cause AKI because hypoalbuminemia leads to decreased oncotic pressure, resulting in a lower effective circulatory volume. The underfilled circulation reduces renal blood flow and precipitates AKI [23]. An animal study demonstrated that a serum albumin level of less than 30 g/L decreased renal perfusion and the glomerular filtration rate [10]. Apart from maintaining the plasma volume with osmotic pressure, albumin also provides many physiological effects, including coupling and carrying various endogenous and exogenous toxic substances, scavenging free radicals, maintaining capillary membrane permeability, providing a physiological reservoir of nitric oxide, imparting an anti-inflammatory effect, and inhibiting apoptosis [24]. The antioxidative properties of albumin help prevent renal tubular cell damage [25, 26], and albumin can improve the survival of cultured renal tubular cells in in vitro settings [26]. Albumin infusion improves AKI in patients with cirrhosis by increasing renal perfusion [27]. However, the benefit of albumin infusion in the prevention of post-LT AKI requires further investigation.

Contrary to adult studies [8, 10], we found no association between hypoalbuminemia and other poor short-term LT outcomes in children in terms of 30-day mortality, length of PICU and hospital stays, duration of mechanical ventilation, need for vasopressors, use of diuretics, persistent drainage, massive ascites, intra-abdominal infection, septicemia, and vascular complications. One hypothesis could be that factors other than serum albumin levels may play a more significant role in determining post-transplant outcomes in this population. For example, the severity of underlying liver disease, perioperative management strategies, and individual patient characteristics may also influence the outcomes.

We also found that patients with AKI had a significantly longer operative time than patients without AKI. The longer operative time may reflect the complexity of the operation, which may in turn affect the effective circulatory volume. However, the estimated blood loss and intraoperative net fluid balance were not significantly different between the two groups (patients with and without AKI).

This study had some limitations. First, this was a retrospective study performed in a single center that mostly performs living-related donor LT; thus, the study population may not represent the overall population of pediatric LT recipients. A large prospective randomized controlled study is needed to investigate the benefit of maintaining normal serum albumin level post-LT. Second, we used the KDIGO criteria to define AKI in this study; however, these criteria may not be sensitive enough, especially in young children. The use of noninvasive biomarkers, such as neutrophil gelatinase-associated lipocalin, may help in the early detection of perioperative AKI in future research. Lastly, the disparity in the number of patients between the low and normal albumin groups, which may have limited the power to detect significant differences in other outcomes.

## Conclusion

Our study highlights the association between postoperative hypoalbuminemia and AKI in pediatric LT recipients. We recommend closer monitoring of serum albumin levels postoperatively and consideration of albumin infusion to maintain intravascular fluid balance which potentially reduce the risk of AKI. Further research is needed to determine the optimal timing and dosage of albumin infusion. These findings suggest actionable steps for clinical practice to improve outcomes in this population.

## Data Availability

The data that support the findings of this study are not openly available due to reasons of sensitivity and are available from the corresponding author upon reasonable request.

## Abbreviations

AKI: Acute kidney injury
IQR: Interquartile range
KDIGO: Kidney Disease: Improving Global Outcomes
LT: Liver transplantation
PELD: Score Pediatric End-Stage Liver Disease score
PICU: Pediatric intensive care unit
POD: Postoperative day
